# Acute compartment syndrome in *Bothrops atrox* envenomation: a case-control study in the Brazilian Amazon

**DOI:** 10.1590/1678-9199-JVATITD-2025-0039

**Published:** 2026-01-30

**Authors:** Glenda de Oliveira Batista do Nascimento, Débora Nery Oliveira, Talia de Oliveira Mota, Suelen Oliveira, André Sachett, Alexandre Vilhena Silva, Wuelton Monteiro, Jacqueline de Almeida Gonçalves Sachett

**Affiliations:** 1 School of Health Sciences, State University of Amazonas, Manaus, AM, Brazil.; 2 Department of Teaching and Research, Dr. Heitor Vieira Dourado Tropical Medicine Foundation, Manaus, AM, Brazil.; 3 Hospital and Emergency Unit 28 de Agosto, State Health Department, Manaus, AM, Brazil.

**Keywords:** *Bothrops* envenomation, Acute compartment syndrome, Risk factors, Amazon

## Abstract

**Background:**

*Bothrops* snakebite is common in the Amazon region and can lead to severe complications in the affected limb, including secondary bacterial infections, blisters, necrosis, and acute compartment syndrome (ACS) in extreme cases. Many of these patients reside in remote areas with limited resources, where early recognition of clinical indicators is decisive for the timely identification of ACS and subsequent decision-making by healthcare professionals. The aim of this study was to identify risk factors associated with ACS following *Bothrops atrox* envenomation in the Brazilian Amazon.

**Methods:**

A case-control study was conducted in three health units of Manaus, Western Brazilian Amazon. The allocation ratio was 1:3, with cases defined as *B. atrox*-envenomed patients developing ACS, and a control group consisting of patients who did not develop ACS.

**Results:**

A total of 37 ACS cases and 111 controls were included in the study. Living in rural areas [OR = 4.59 (95%CI = 1.51-20.0; p = 0.017)], bites in the lower limbs [OR = 7.6 (95%CI = 3.18-19.3; p < 0.001)], time to medical care of 7-12 hours [OR = 4.23 (95%CI = 1.63-11.1; p = 0.003)], blisters [OR = 3.24 (95%CI = 1.12-9.25; p = 0.027)], and secondary bacterial infection [OR = 15 (95%CI = 3.54-103; p < 0.001)] were associated with ACS. Mean values of creatine kinase were significantly higher in ACS patients on the first (p = 0.022) and second (p = 0.013) days of hospitalization.

**Conclusion:**

This study presents, for the first time, the factors associated with ACS from *B. atrox* envenomation, providing a basis for early diagnosis and treatment, and enabling prompt medical intervention. This may reduce adverse events, promote faster recovery, and lower the rate of disability.

## Background

Snakebite envenomations (SBEs) caused by the genus *Bothrops* constitute an important public health problem in Latin America and some countries of the Caribbean [1]. The venom of *Bothrops* causes a series of changes in the bitten limb, resulting from the direct effect of toxins producing tissue damage, changes in blood flow to the site associated with coagulation disorders, and an associated acute inflammatory process [2-4]. In humans, relevant pathological changes are observed in all strata of the skin, with an emphasis on hemorrhage, inflammatory infiltrate, edema, congestion, and vascular damage [5]. Venom-induced consumption coagulopathy is a hallmark of local and systemic envenomation, resulting in ischemic and hemorrhagic manifestations [6, 7]. Local complications such as secondary infections, blisters, and necrosis are relatively common [2, 8, 9]. Even though less common, patients can develop compartment syndrome, which, if not treated timely, may cause severe long-term disabilities [10-12]. 

Acute compartment syndrome (ACS) is a severe complication resulting from snakebites. This complication is characterized by an increased pressure in one or more muscle compartments, which consequently reduces capillary perfusion, leading to ischemia and subsequent tissue damage, requiring urgent treatment [13, 14]. The progressive increase in intracompartment pressure in the closed osteofacial space combined with edema in soft tissues, exceeding venous and arterial pressure, generates tissue ischemia and, when not treated in a timely manner, can progress to necrosis and limb loss [15, 16]. The recommended treatment for compartment syndrome is fasciotomy surgery, used to reduce pressure in the compartment of the affected limb [17].

Associated factors for ACS are known for different types of trauma, such as forearm fractures [18], supracondylar humerus fractures [19], tibial fractures [16, 20, 21], and foot fractures [22]. The public hospital cost for treating snakebite is considerable [23], and when related to ACS, it can have a high cost of treatment due to complex procedures and longer hospitalization periods, representing a 2.3-fold increase in the cost compared to patients without this complication [24, 25]. 

Early diagnosis with immediate intervention can modify the unfavorable prognosis, reducing the disability rate [26, 27]. However, in regions such as the Amazon, there are challenges such as geography and logistics that can contribute to late diagnosis and subsequent delayed treatment, which can lead to the worse outcomes. Furthermore, the factors associated with ACS occurring as a complication of snakebites are not known; identifying these factors could enhance clinical decision-making, early detection, and intervention, improving patient outcomes and care quality.

The aim of this study was to identify factors associated with compartment syndrome from *Bothrops atrox* envenomations in the Brazilian Amazon.

## Methods

### 2.1 Study site

The state of Amazonas, with a vast territorial area of 1,570,946.8 km² and 62 municipalities, combined with a rich cultural diversity, faces unique health challenges in providing high-complexity care. Despite progress in decentralizing healthcare services, many municipalities still rely on Manaus, the capital, for specialized care, highlighting regional disparities in the availability of advanced medical services. Hospitals in the interior of Amazonas are generally equipped for primary and medium-complexity care. However, for complications of snakebite envenomation requiring specialized interventions, such as extensive fasciotomies for compartment syndrome, dialysis for renal failure, and intensive care for severe cases, patients are often transferred to Manaus. These cases are managed in major facilities in the capital, such as the Dr. Heitor Vieira Dourado Tropical Medicine Foundation (FMT-HVD), the 28 de Agosto Emergency Hospital (HPS 28 de Agosto), and the Children’s Emergency Hospital in the Eastern Zone, which served as study sites for this research.

### 2.2 Study design

This study was designed as a case-control investigation aiming to identify factors associated with ACS in patients envenomed by *Bothrops* treated at three healthcare units in Manaus, Brazilian Amazon: Fundação de Medicina Tropical Dr. Heitor Vieira Dourado (FMT-HVD), the 28 de Agosto Emergency Hospital (HPS 28 de Agosto), and the Children’s Emergency Hospital of the Eastern Zone. These facilities were selected for their status as orthopedic reference centers performing fasciotomy procedures in ACS cases.

The control group consisted of patients treated exclusively at the FMT-HVD, in a 1:3 ratio, and included individuals diagnosed with *B. atrox* envenomation who did not develop ACS. These patients were selected based on admissions occurring on dates subsequent to those of the cases included in the study. The case group comprised patients diagnosed with ACS following *Bothrops* envenomation, based on clinical criteria such as: edema, pain, pallor, pulselessness, paresthesia, paralysis, and poikothermia [17, 28].

The variables studied included sociodemographic data such as sex, age, and place of residence; epidemiological data such as snakebite severity classification, pre-hospital care, time elapsed from snakebite to medical care, local and systemic manifestations, time from hospital admission to ACS diagnosis, clotting time (CT) and creatine kinase (CK) levels. 

On admission, severity classification was carried out in accordance with the Brazilian Ministry of Health protocol [29]:

- *mild*: minor local manifestations,
- *moderate*: edema and bruising, and minor systemic bleeding,
- *severe*: severe local manifestations, severe bleeding, and shock.

### 2.3 Data source

Data collection for both the control and case groups at FMT-HVD was conducted through a retrospective review of physical and electronic medical records (iDoctor). ACS patients from the 28 de Agosto Hospital were identified through daily census records from the surgical center and from electronic medical records (Medview). At the Children’s Emergency Hospital of the Eastern Zone, ACS cases were identified via the Patient Records Service, which manages physical records for this unit. Data collection covered the period from May 2022 to January 2024.

### 2.4 Data analysis

The data were analyzed using R software v. 4.2 and R Studio v. 2023.3. The proportions of severe cases were compared using the Chi-square test or Fisher’s exact test, when appropriate. The crude odds ratio (OR) with its 95% confidence interval (CI) was calculated considering the occurrence of compartment syndrome as the dependent variable. Mean and standard deviation were used to describe continuous variables, which were compared using univariate logistic regression test. Statistical significance was considered when p < 0.05.

### 2.5 Ethical clearance

The research was approved by the Research Ethics Committee of the State University of Amazonas under the CAAE number 05541618.3.0000.5016. Written informed consent was obtained from the patients for the publication of the case details and accompanying images.

## Results

A total of 37 ACS cases and 111 controls were included in this study. 

### ACS patients’ characteristics

Of the 37 evaluated patients who presented with ACS, the following epidemiological characteristics were observed: 67.57% (25/37) were aged between 14 and 60 years; 83.78% (31/37) were male; and 91.89% (34/37) lived in rural areas. Concerning the envenomations, 54.05% (20/37) were initially classified as moderate, 86.11% (31/37) of the bites were on the lower limbs or foot, and in 66.44% (24/37) of the cases, the time elapsed between the accident and medical care exceeded seven hours (Table 1).

**Table 1.** Acute compartment syndrome in *Bothrops atrox* snakebites.

Characteristic	Overall n = 148, (%)	Control group n = 111, (%)	Case group n = 37, (%)	Univariate		p
				OR	CI95%	
Age (years)						
< 14	28/148 (18.92)	17/111 (15.32)	11/37 (29.73)	-	-	
14-30	42/148 (28.38)	32/111 (28.83)	10/37 (27.03)	0.48	0.17, 1.36	0.200
31-60	67/148 (45.27)	52/111 (46.85)	15/37 (40.54)	0.45	0.17, 1.17	0.100
> 60	11/148 (7.43)	10/111 (9.01)	1/37 (2.70)	0.15	0.01, 0.98	0.095
Area of occurrence						
Urban	35/148 (23.65)	32/111 (28.83)	3/37 (8.11)	-	-	
Rural	113/148 (76.35)	79/111 (71.17)	34/37 (91.89)	4.59	1.51, 20.0	0.017
Sex						
Male	120/148 (81.08)	89/111 (80.18)	31/37 (83.78)	-	-	
Female	28/148 (18.92)	22/111 (19.82)	6/37 (16.22)	0.78	0.27, 2.01	0.600
Severity classification on hospital admission						
Severe	38/146 (26.03)	21/109 (19.27)	17/37 (45.95)	-	-	
Moderate	72/146 (49.32)	52/109 (47.71)	20/37 (54.05)	0.48	0.21, 1.08	0.076
Mild	36/146 (24.66)	36/109 (33.03)	0/37 (0.00)	0	-	> 0.900
Time from bite to hospital care (hours)						
0 a 6	89/143 (62.24)	74/107 (69.16)	15/36 (41.67)	-	-	
7 a 12	26/143 (18.18)	14/107 (13.08)	12/36 (33.33)	4.23	1.63, 11.1	0.003
> 12	28/143 (19.58)	19/107 (17.76)	9/36 (25.00)	2,34	0.87, 6.12	0.086
Tourniquet use	18/148 (12.16)	15/111 (13.51)	3/37 (8.11)	0,56	0.13, 1.84	0.400
Anatomical region of the bite						
Foot	86/147 (58.50)	76/111 (68.47)	10/36 (27.78)	-	-	
Lower limbs	42/147 (28.57)	21/111 (18.92)	21/36 (58.33)	7.60	0.75, 8.95	< 0.001
Upper limbs	19/147 (12.93)	14/111 (12.61)	5/36 (13.89)	2.71	3.18, 19.3	0.110
Extent of edema (limb segments)						
1	3/32 (9.38)	2/16 (12.50)	1/16 (6.25)	-	-	
2	2/32 (6.25)	2/16 (12.50)	0/16 (0.00)	0		> 0.900
3	11/32 (34.38)	6/16 (37.50)	5/16 (31.25)	1.67	0.12, 42.4	0.700
5	16/32 (50.00)	6/16 (37.50)	10/16 (62.50)	3.33	0.26, 81.4	0.400
Days of hospitalization						
1-3	67/141 (47.52)	53/108 (49.07)	14/33(42.42)	-	-	
4-7	41/141 (29.08)	31/108 (28.70)	10/33(30.30)	1.22	0.47, 3.07	0.700
8-14	23/141 (16.31)	19/108 (17.5)	4/33 (12.12)	0.80	0.21, 2.55	0.700
> 15	10/141 (7.09)	5/108 (4.63)	5/33 (15.15)	3.79	0.94, 15.5	0.057
Local manifestations						
Erythema	32/144 (22.22)	27/111 (24.32)	5/33 (15.15)	0.56	0.18, 1.48	0.300
Bleeding	37/147 (25.17)	29/111 (26.13)	8/36 (22.22)	0.81	0.31, 1.91	0.600
Ecchymosis	22/146 (15.07)	18/111 (16.22)	4/35 (11.43)	0.67	0.18, 1.95	0.500
Blisters	17/147 (11.56)	9/111 (8.11)	8/36 (22.2)	3.24	1.12, 9.25	0.027
Secondary infection/local inflammatory response syndrome	10/148 (6.76)	2/111 (1.80)	8/37 (21.62)	15.00	3.54, 10.3	< 0.001
Necrosis	32/144 (22.22)	5/111 (4.50)	4/37 (10.81)	2.57	0.61, 10.3	0.200
Systemic manifestations						
Acute renal failure	11/148 (7.43)	8/111 (7.21)	3/37 (8.11)	1.14	0.24, 4.18	0.900
Vomiting	19/147 (12.93)	17/111 (15.32)	2/36 (5.56)	0.33	0.05, 1.22	0.150
Syncope	4/147 (2.72)	3/111 (2.70)	1/36 (2.78)	1.03	0.05, 8.33	> 0.900
Fever	20/147 (13.61)	13/111 (11.71)	7/36 (19.44)	1.82	0.63, 4.89	0.200
Hematuria	4/147 (2.72)	2/111 (1.80)	2/36 (5.56)	3.21	0.37, 27.5	0.300
Nausea	19/147 (12.93)	17/111 (15.32)	2/36 (5.56)	0.33	0.05, 1.22	0.150
Gingival bleeding	11/148 (7.43)	6/110 (5.45)	3/36 (8.33)	1.58	0.32, 6.33	0.500
Lee-White clotting time*						
Normal	63/128(49.22)	51/108 (47.22%)	12/20(60.00%)	-	-	
Incoagulable	65/128(49.22)	57/108 (52.78%)	8/20 (40.00%)	0.67	0.22, 1.56	0.300

OR: odds ratio; CI: confidence interval.

*Reference values for Lee-White clotting time: 10 minutes.

The diagnosis of ACS was established within the first hours after the snakebite in 21 patients (56.76%), within 24 hours in ten patients (27.03%), and after two or more days in six patients (16.21%). None of these cases was classified as mild envenomation, and 26 patients had an indication for antivenom administration based on the clinical assessment of the envenomation.

Of the 37 patients, ten (27.02%) underwent mechanical-surgical cleaning to remove areas of necrosis and devitalized tissue, 17 (45.95%) incisions were made on the lower limbs, and 5 (13.51%) had their surgical wounds covered with polypropylene plastic.

The following traditional procedures performed by victims (or their families) at the bite site in the pre-hospital setting were registered: use of *Aloe vera* leaves, alcoholic beverages, gasoline, alcohol, paca powder, wood sawdust, skin from the snake responsible for the bite, boiled eggs, and lip suction.

### 3.2 Associated factors for ACS in *Bothrops atrox* snakebites

Living in rural areas [OR = 4.59 (95%CI = 1.51-20.0; p = 0.017)], bites in the lower limbs [OR = 7.6 (95%CI = 3.18-19.3; p < 0.001)], time to medical care of 7-12 hours [OR = 4.23 (95%CI = 1.63-11.1; p = 0.003)], blisters [OR = 3.24 (95%CI = 1.12-9.25; p = 0.027)], and secondary bacterial infection/local inflammatory response syndrome [OR = 15 (95%CI = 3.54-103; p < 0.001)] were associated with ACS (Table 1).

**Figure 1.**
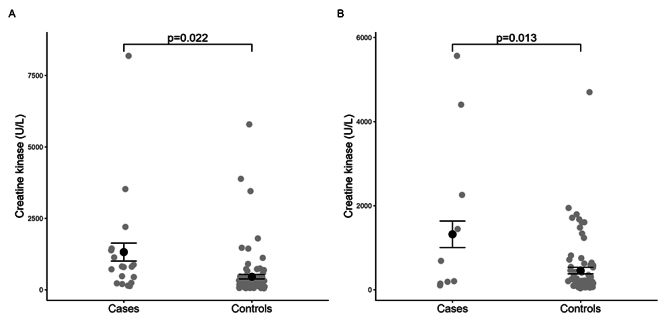
Comparison of creatine kinase activity values between cases and controls on **(A)** the first day and **(B)** the second day of hospitalization.

Mean creatine kinase activity values were significantly higher in ACS patients on the first (p = 0.022) and second (p = 0.013) days of hospitalization (Figure 1).

### 3.3 Cases follow-up

**Figure 2.**
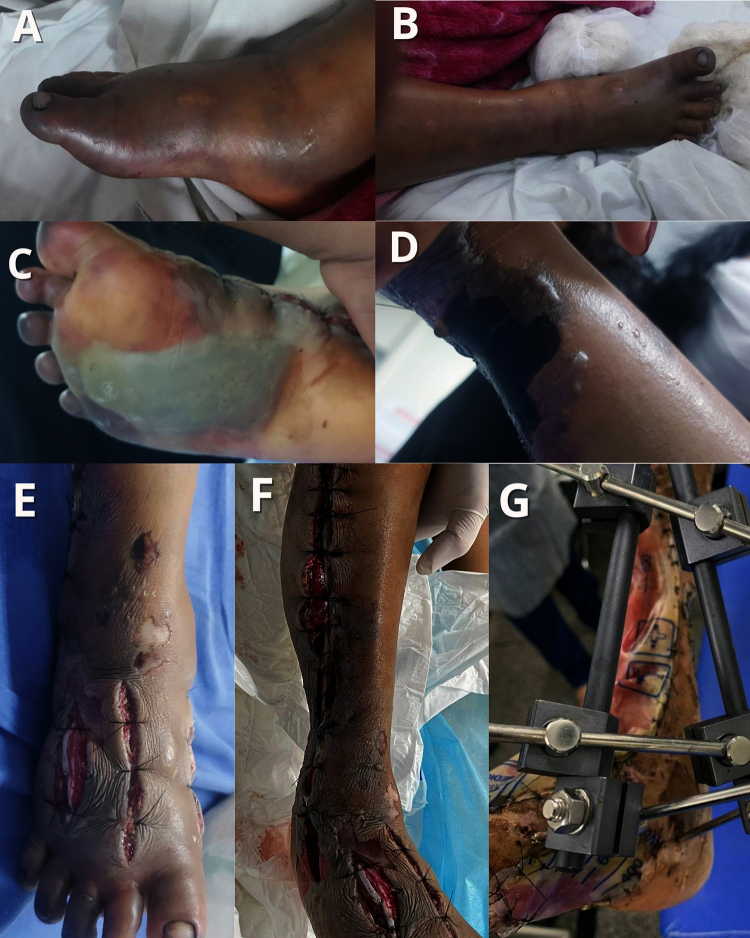
A 10-year-old male patient, victim of *Bothrops* envenomation with multiple bites to the right lower limb. **(A, B)** At the time of hospital admission, the patient presented significant edema and signs of ACS. According to clinical history, hospitalization occurred more than 30 hours after the snakebite, with the patient already in critical condition. **(C, D)** The patient developed severe complications, such as extensive blisters and areas of necrosis, and underwent fasciotomy of the right leg and foot, as well as **(E, F)** surgical debridement with significant tissue loss and tendon exposure. The surgical wound was covered with polypropylene plastic from a sterile urine collection bag, and **(G)** external fixation of the affected limb was applied. Following these procedures, amputation of the 3^rd^, 4^th^ and 5^th^ right toes was performed. After 40 days of hospitalization, the patient was discharged.

**Figure 3.**
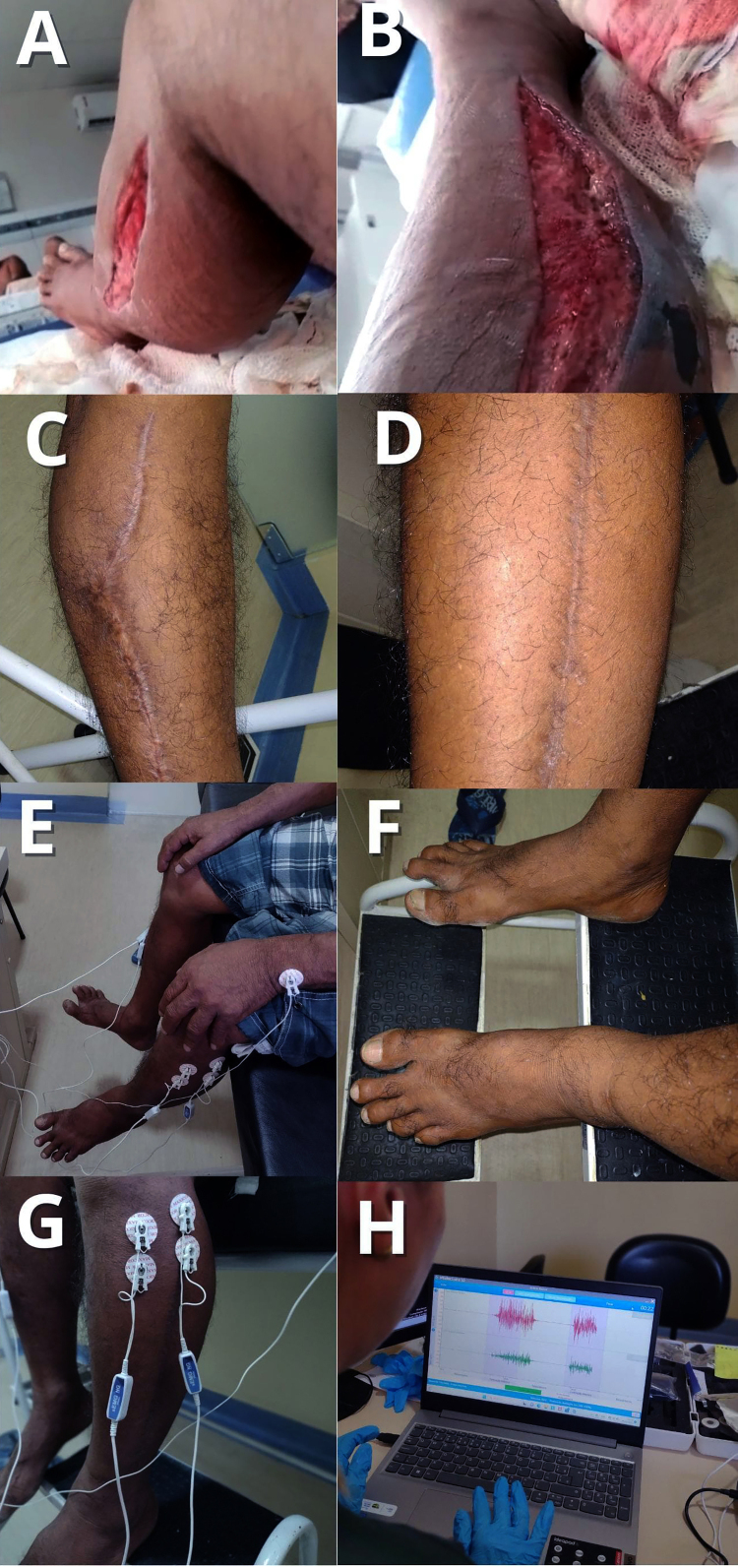
A 59-year-old male patient evaluated 4 months after hospital discharge. The patient was a victim of a *Bothrops* envenomation on the left lower limb, presenting local injury to the affected limb, pain, edema, blisters, initially classified as moderate edema, numbness, tingling, coagulation time of 8 minutes, and CK of 879 U/L. In his hometown, after the snakebite, he reported squeezing the bite site and driving for 29 minutes to the hospital where antivenom therapy with six vials of *Bothrops* antivenom was administered. **(A)** He underwent fasciotomy surgery due to progression to ACS, **(B)** presenting necrosis at the bite site, and surgical debridement was performed. The patient progressed to a severe case of acute renal failure and secondary infection, requiring prolonged ICU hospitalization. **(C, D)** In an interview, he reported that some sutures at the bite site ruptured after the surgical wound was closed, but the wound showed good healing on both sides of the affected limb. Currently, he presents gait alteration, **(E)** constant pain around the ankle, pain on exertion near the scar (bite site), and difficulty performing work and daily activities. **(E)** An evaluation with myoelectrostimulation was performed, using electrodes to measure muscle strength. **(F)** A slight bone deviation and edema in the affected limb were observed, but no pain was reported, with paresthesia above the heel and **(G, H)** a considerable decrease in muscle strength in response to stimulation when compared to the contralateral limb, where the affected limb is represented in green and the contralateral limb in red.

**Figure 4.**
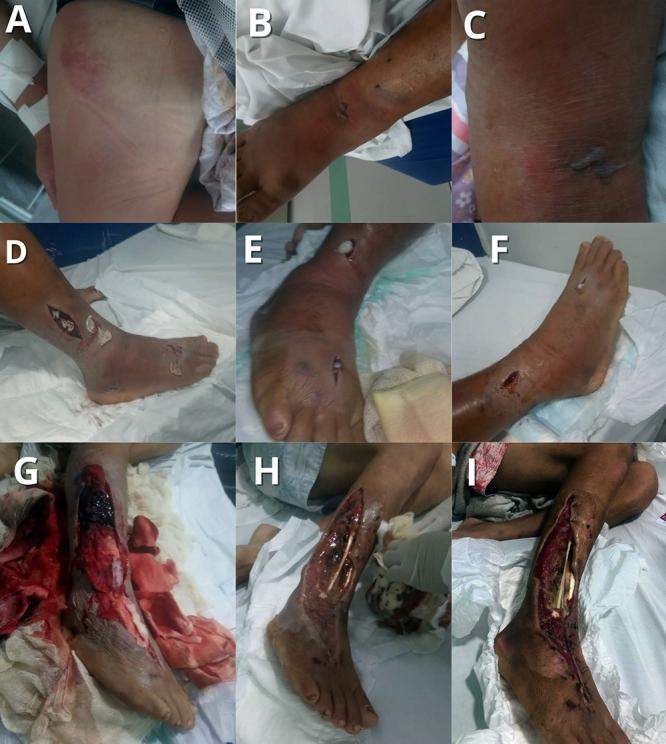
A 33-year-old male patient, evaluated during hospitalization. The patient suffered a *Bothrops* envenomation, classified as severe, and used 16 vials of *Bothrops* antivenom. During hospitalization, he presented significant **(A)** edema and **(B, C)** blisters, **(D, F)** as well as an abscess that was drained multiple times in the affected area, progressing to ACS, **(D, F)** where multiple initial incisions were made to decompress the limb. **(G)** Later, the incisions were enlarged for more effective decompression, with significant regression of the edema and drainage of seropurulent secretion. **(H, I)** The patient exhibited bone and tendon exposure, with the onset of healing and the formation of granulation tissue, showing CK levels of 2725 U/L and a coagulation time of 9 minutes. The patient was transferred to another hospital unit for skin grafting.

**Figure 5.**
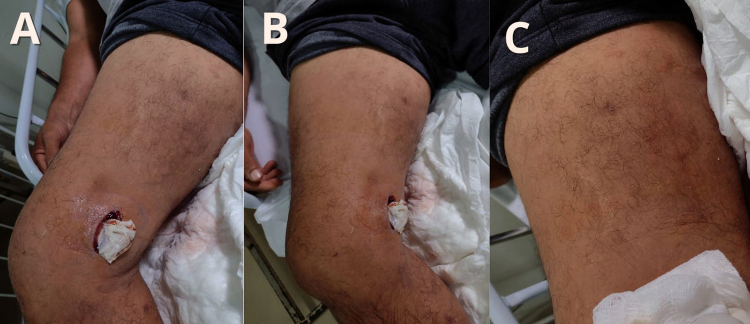
A 48-year-old male patient, evaluated during hospitalization. Victim of *Bothrops* envenomation on the right lower limb. During treatment, the patient had an adverse reaction to the antivenom, using 12 vials of *Bothrops* antivenom. Laboratory results showed a coagulation time of 10 minutes and CK of 147 U/L. The patient was diagnosed with ACS and developed a secondary infection. **(A-C)** He presented with a hardened bruising area near the knee and underwent a small incision for decompression of the affected limb, progressing with improvement and hospital discharge.

In four patients with ACS, follow-up was conducted prospectively, allowing for evaluations both during hospitalization and after discharge. This approach made it possible to monitor the clinical progression, the healing of surgical wounds, and the existence of possible physical or functional sequelae (Figures 2-5). 

## Discussion

The development of complications such as ACS in the context of snake envenomation by *B. atrox* has been scarcely reported in the general population [9, 30], and even less so in Indigenous populations [11]. Some cases of ACS described in the literature also involve envenomations by *B. jararaca* [31]*, B. asper* [3, 32, 33] and *B. jararacuçu* [34]. Thus, this study aimed to understand the relationship between envenomation and risk factors for the development of ACS in patients bitten by *Bothrops* snakes.

Local effects in *Bothrops* envenomation, such as ecchymosis, abscesses, blisters, necrosis, and compartment syndrome, are not uncommon and often require specific medical procedures due to the presence of tissue complications [8, 35-37]. Among these complications, compartment syndrome is considered the most severe local manifestation, as it can lead to extensive tissue necrosis, ischemia, and neuropathy, in addition to functional sequelae that may result in limb amputation [38]. Compartment syndrome due to snake envenomation can also be considered a combined trauma, involving soft tissue injuries with secondary vascular repercussions [39, 40]. In addition, depending on its severity, muscle necrosis can cause myoglobinemia, which may contribute to the development of systemic complications such as acute kidney injury [41]. In this study, risk factors for ACS in *Bothrops* envenomation included snakebite occurrence in rural areas, lower limb bites, arrival at a healthcare facility more than six hours after the incident, elevated creatine kinase levels on the first and second days of hospitalization, concurrent secondary infection, and the presence of blisters on the affected limb from the first day of envenomation. Additionally, patients over 60 years old showed a protective trend.

Several associated clinical signs may increase the risk of local complications, such as local inflammatory response syndrome (LIRS) and secondary bacterial infection, which, following snakebite envenomation, represent major clinical and scientific challenges. Since the mid-1990s, efforts have been devoted to understanding the immunological mediators involved and recognizing the complexity of venom-induced inflammatory mechanisms[42-46]. The release of pro-inflammatory cytokines such as IL-6 and IL-8 in patients envenomed by snakes of the genera *Bothrops* and *Crotalus* has been characterized as an acute-phase response, with leukocytosis, neutrophilia, and increased levels of acute-phase proteins arising from inflammatory mechanisms intrinsic to envenomation, rather than being exclusively attributable to bacterial infection[42]. TNF-α, involved in the pathogenesis of local necrosis, is released following venom-induced metalloproteinase activity, thereby triggering necrosis and perpetuating inflammation. This underscores the importance of early treatment, as antivenom has limited efficacy against established necrosis [45].

*Bothrops atrox* venom induces an early increase in vascular permeability, leukocyte influx, and the release of multiple inflammatory mediators (cytokines, eicosanoids, and chemokines), clinically triggering local inflammatory response syndrome, which may mimic bacterial infection and make differential diagnosis even more challenging [46]. Venom metalloproteinases and C-type lectins also contribute to local and systemic inflammatory processes, creating an intersection between the innate immune and hemostatic systems, with overlapping inflammation and coagulation disturbances that further complicate the clinical response in *Bothrops* envenomation. Alongside these local effects, fever emerges as an acute-phase clinical marker, often misinterpreted as infection but in fact reflecting the intrinsic inflammatory response to envenomation [43]. These observations carry practical implications, as the indiscriminate use of antibiotics in patients without confirmed infection may contribute to antimicrobial resistance without providing clinical benefit.

The incidence of ACS is high in the diaphysis, particularly in long muscles such as those of the lower limbs (which comprise four muscle compartments: anterior, lateral, deep posterior, and superficial posterior), as observed in the patients in this study. This occurs due to the anatomy of these segments, which are surrounded by larger muscle bellies that limit significant expansion caused by extensive edema [47], making soft tissue damage a prevalent predictor and cause of leg ACS [48]. Furthermore, elevations in creatine kinase (CK) suggest muscle breakdown due to ischemia, damage, or rhabdomyolysis [47, 49]; our study demonstrated an association between high levels of this enzyme and the development of ACS on the first day of *Bothrops* envenomation and also on the following day. Inflammatory markers, such as increased WBC count and AST levels, may also serve as adjuncts in clinical and laboratory assessments, potentially indicating an inflammatory or cytokine reaction following a severe snakebite [50].

Understanding risk factors can improve the accuracy of ACS diagnosis, which remains a clinical challenge, especially since healthcare facilities do not always have specialized tools for early recognition of the condition. In remote regions such as the Amazon, the structural limitations of healthcare services, the lack of equipment, and the shortage of orthopedic specialists in distant areas [51] make clinical assessment tools particularly valuable for early decision-making and reducing disabilities in affected patients.

Historically, clinical diagnosis has relied on the "6 P’s"- pain, pallor, poikilothermia, paresthesia, paralysis, and pulselessness - as early indicators of ACS, with particular attention given to disproportionate and exacerbated pain in the muscle compartments near the bite site [17, 52]. Classically, when these clinical symptoms are present alongside a pressure ﻿differential ΔP ( ﻿30 mmHg (ΔP = diastolic pressure - intracompartmental pressure) [53], surgical decompression should be performed within one hour [54]. However, a single normal intracompartmental pressure measurement may not exclude acute compartment syndrome [47, 49, 52]. On the other hand, non-invasive techniques can also aid in the diagnostic assessment of ACS. In a study conducted in Taiwan with 63 patients, while the "6 P’s" were used as surgical predictors, the presence of Doppler flow was identified as a clinical indicator against performing fasciotomy, suggesting that patients with persistent blood flow detected by ultrasound, despite local signs and symptoms, may not require the procedure [55]. Furthermore, the progression of edema in snakebite envenomation can be monitored via ultrasound, allowing for early non-invasive diagnosis and timely indication for fasciotomy when necessary [56]. Pharmacological interventions can also be employed prophylactically in the early stages of ACS to reduce edema and control reperfusion injury [57, 58].

The early diagnosis and treatment of ACS are essential to prevent severe long-term disability, with a critical window for performing fasciotomy within eight hours from ACS diagnosis [53, 59, 60]. Longer periods of ischemia related to ACS are correlated with worse outcomes [53, 55]. Muscles can typically tolerate 6 to 8 hours of ischemia before necrosis occurs. However, this timeframe may vary depending on the extent of trauma, the amount of venom injected, and the volume of the affected muscle groups [59, 61-63]. In some cases, muscle necrosis can develop as early as three hours after injury [64]. 

Compartment ischemia due to arterial injury initiates a vicious cycle, leading to increased pressure, often exacerbated by limb reperfusion [59, 65, 66]. In snake envenomation, compartment syndrome most often develops within the first 24 hours [50]. In the *Bothrops* envenomations analyzed in this study, 83.78% of ACS cases occurred within 24 hours of the bite. Early access to healthcare services and administration of antivenom within six hours were identified as protective factors against ACS development. However, after this period, ACS can still occur, with reports documenting its presence in 22.2% of cases [50]. Therefore, patients should be closely monitored for at least 48 hours post-envenomation to ensure early detection and management of this local complication.

During ACS development, muscle revascularization after a period of ischemia can lead to a further increase in intracompartmental pressure, often necessitating fasciotomy [47, 59]. In cases where muscle injury is associated with vascular injuries, a high incidence of ACS with an increased rate of fasciotomies may occur, especially when both the artery and vein are affected [40, 63, 65, 67-70]. Conversely, some clinicians may opt for prophylactic fasciotomies to prevent reperfusion ischemia and the onset of ACS [71]. There is no precisely defined timeframe after which irreversible muscle damage occurs [53] , and the actual incidence remains unclear, as it depends on a broad and potentially variable spectrum of clinical presentations [61-63]. 

Delayed diagnosis of snakebite and late venom neutralization with antivenom can occur for various reasons, with the distinct therapeutic itinerary in the Amazon being a common reality. Even when patients seek immediate medical attention after envenomation, it may take days for them to reach the first healthcare facility [72]. This delay often exceeds six hours before antivenom administration, identifying time as a significant risk factor in this study and contributing to a higher incidence of ACS compared to other regions. Furthermore, delays in clinical decision-making by healthcare professionals or logistical challenges can negatively impact functional outcomes, leading to impaired daily activities due to muscle dysfunction, disabilities, and severe complications, including tissue and nerve damage, gangrene, and amputation [73, 74]. The time-effect relationship regarding delayed ACS treatment in snake envenomation remains unclear [73], but prolonged delays may result in permanent injuries, leading to long-term disabilities ranging from sensory loss to limb amputations, ultimately compromising quality of life [11, 12, 30, 75].

Even with the timely identification of ACS and the implementation of procedures such as fasciotomy, the patient is not exempt from risks. Therefore, the risk-benefit balance should always be considered in the best interest of the patient [73]. Muscle regeneration in patients with snake envenomation, which may be aggravated by extensive invasive procedures as described in the cases of this study, may not fully occur due to myonecrosis that damages muscle fibers, nerves, and the microvasculature, with this tissue being replaced by fibrosis [33]. This is related to the venom’s activity, the inflammatory response, or the presence of an infectious process, or both [5]. 

A limitation of this study is that clinical evaluation of ACS with conventional diagnosis through intracompartmental pressure measurement is not routinely available within the Brazilian public health service, and only clinical evaluation for ACS diagnosis was used. Furthermore, patient hospitalization records sometimes did not contain all data related to snakebite and/or ACS, which resulted in missing data for some variables.

## Conclusion

This study is the first to identify factors associated with ACS resulting from *Bothrops atrox* envenomation, providing a basis for future clinical research on treatment and rehabilitation. The development of acute compartment syndrome (ACS) was primarily associated with delayed medical attention, bites to the lower limbs, elevated creatine kinase levels, secondary infection, blisters, and being in the economically active age group (engaged in forest-related activities). Geographic barriers and long travel times further contributed to this outcome. Post-discharge monitoring is essential, particularly in ACS cases, to track complications, assess disabilities, and ensure integration into rehabilitation services. 

## Availability of data and materials

 All data generated or analyzed during this study are included in this article.
